# Influence of transcatheter aortic valve replacement on patients with severe aortic stenosis undergoing non-cardiac surgery

**DOI:** 10.1186/s13019-020-01237-5

**Published:** 2020-07-29

**Authors:** Tadashi Omoto, Atsushi Aoki, Kazuto Maruta, Tomoaki Masuda, Yui Horikawa

**Affiliations:** Department of Cardiovascular Surgery, Showa University, Hatanodai 1-5-8, Shinagawa-ku, Tokyo, 142-8666 Japan

**Keywords:** Aortic stenosis, Non-cardiac surgery, Transcatheter aortic valve replacement

## Abstract

**Objectives:**

The purpose of this study was to clarify the influence of transcatheter aortic valve replacement (TAVR) in patients with aortic stenosis (AS) undergoing non-cardiac surgery.

**Methods:**

Thirty-four patients with severe AS diagnosed by preoperative evaluation for non-cardiac surgery were reviewed and compared in two categories. First, patient profiles and surgical risk were compared before (pre-TAVR group; *n* = 10) and after (post-TAVR group; *n* = 24) the introduction of TAVR. Second, the completion rate of non-cardiac surgery and interval between the two cardiac and non-cardiac operations were compared between surgical aortic valve replacement (AVR) patients before the introduction of TAVR (pre-AVR group (*n* = 10)), in AVR patients after the introduction of TAVR (post-AVR (*n* = 12)), and in TAVR patients (TAVR group (*n* = 12)).

**Results:**

Age and Japan score were higher in the post-TAVR group than in the pre-TAVR group. Malignancy was the most common non-cardiac disease (80%) in the pre-TAVR group, whereas orthopedic disease was the most common (50%) in the post-TAVR group. Completion rate of non-cardiac operation in the pre-AVR, post-AVR and TAVR groups was 70, 33, and 75% (post-AVR vs. TAVR: *p* = 0.010), and the interval between the two operations was 129 ± 98 days, 87 ± 40 days and 27 ± 15 days, respectively (pre AVR vs. TAVR: *p* = 0.034 and post AVR vs. TAVR: *p* = 0.025). In the post-TAVR group, AVR was selected because of a lack of fitness for TAVR in 5 of 12 patients.

**Conclusions:**

After the introduction of TAVR, more senile and high-risk patients became candidates for a two-stage operation, and orthopedic conditions became the most common non-cardiac disease. Innovation in transcatheter valvular interventions and expansion of indications for patients currently evaluated as “unfit for TAVR” might be crucial issues for non-cardiac surgery with severe AS.

## Introduction

Preexisting aortic stenosis (AS) is a high risk factor for non-cardiac surgery [1, 2]. Guidelines from the American College of Cardiology and the American Heart Association (ACC/AHA) recommend aortic valve replacement (AVR) before non-cardiac surgery [3]. However, older patients, especially those with numerous co-morbid conditions or frail patients, tend to refuse two successive major operations.

Recently, transcatheter aortic valve replacement (TAVR) emerged as an alternative option for high-risk patients with symptomatic severe AS who are unable to undergo surgical AVR. The purpose of this study was to clarify the influence of the introduction of TAVR on patient background, type of non-cardiac disease, completion rate of non-cardiac surgery and interval between the two operations in patients with AS.

## Subjects and methods

This study complies with the Declaration of Helsinki, and was approved by the Institutional Review Board of Showa Medical University. In this retrospective study, we reviewed 161 patients with severe AS who underwent surgical intervention between April 2011 and May 2019, and enrolled 34 patients with severe AS diagnosed during preoperative evaluation for elective non-cardiac surgery. TAVR was introduced in our institute beginning October 2015. The question of whether to perform surgical AVR or TAVR was discussed by the heart team, consisting of cardiologists and cardiac surgeons.

Aortic valve area, peak flow velocity and mean aortic valve pressure gradient, as well as left ventricular ejection fraction were extracted from the echocardiographic database. Severe AS was defined using current echocardiographic criteria, namely aortic valve area ≤ 1 cm^2^, peak systolic flow velocity ≥ 4 m/s, or mean gradient ≥40 mmHg [4]. Therapeutic options were also discussed for patients with low flow or a low pressure gradient AS, i.e., mean gradient < 40 mmHg and left ventricular ejection fraction ≤40%. Baseline characteristics of patients were collected from medical records, and additional details were obtained from the referring physician by telephone interview.

First, patient profile and surgical risk were compared between pre-TAVR group (*n* = 10); patients before the introduction of TAVR (April 2011–September 2015, *n* = 10) and the post-TAVR group (*n* = 24); and patients after introduction of TAVR (October 2015 ~ May 2019). The Japan score was used as risk score to predict preoperative mortality in Japanese subjects [5]. Second, completion rate of non-cardiac surgery and the interval between the two operations were compared between pre-AVR group (*n* = 10); those who underwent AVR before the introduction of TAVR and the post-AVR group (*n* = 12); those who underwent AVR after the introduction of TAVR and the TAVR group (*n* = 12); and those who underwent TAVR.

Demographic and clinical data were expressed as mean ± standard deviation or number (%). Baseline differences in categorical variables were tested using the Pearson χ^2^ test, while analysis of variance was used for comparing differences in means among the groups. Values of *p* < 0.05 were considered statistically significant. Statistical analysis was performed using JMP Pro 13 (SAS Inc., NC, USA).

## Results

### Pre-TAVR vs. post-TAVR

**Fig. 1:**
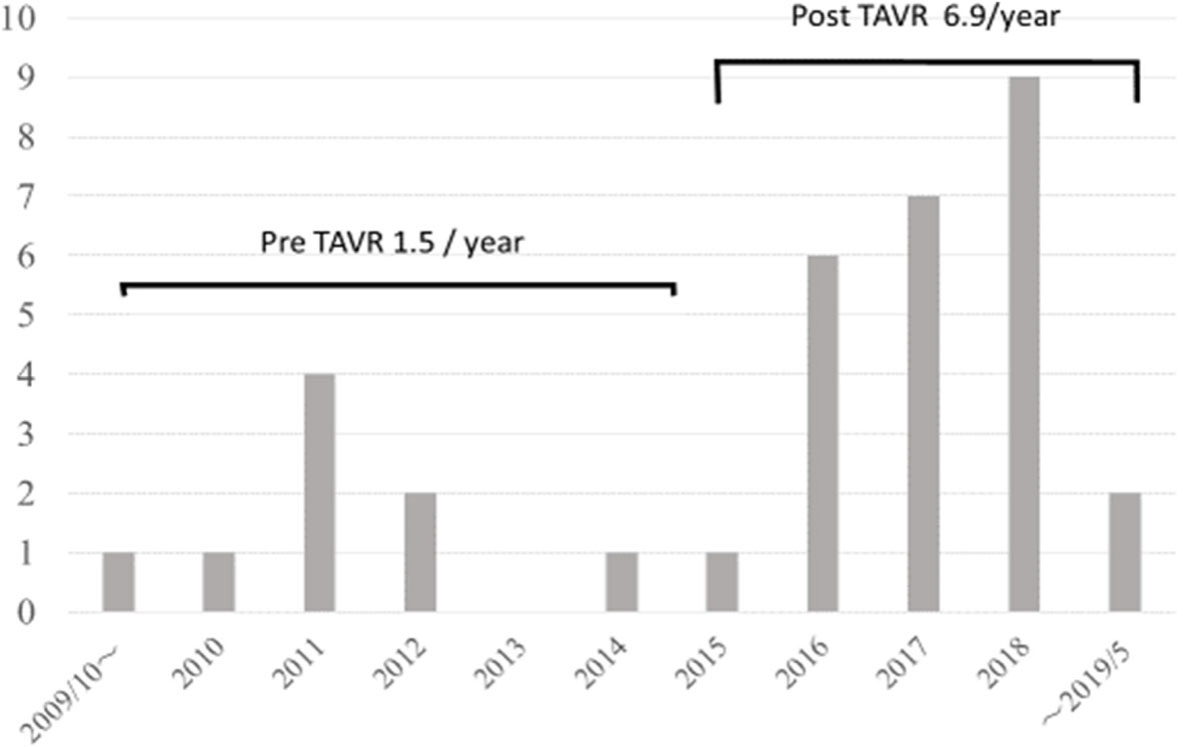
Annular number of patients who consulted the Department of Cardiovascular Surgery due to severe aortic valve stenosis diagnosed at preoperative evaluation

The annual number of patients who underwent AVR or TAVR due to severe AS was 20.9 cases/year before the introduction of TAVR and 18.6 cases/year after introduction. The annual number of patients who consulted the Department of Cardiovascular Surgery due to severe AS diagnosed on preoperative evaluation of non-cardiac disease in these respective time periods was 1.5 and 6.9 cases/year, respectively (Fig. 1).

**Fig. 2:**
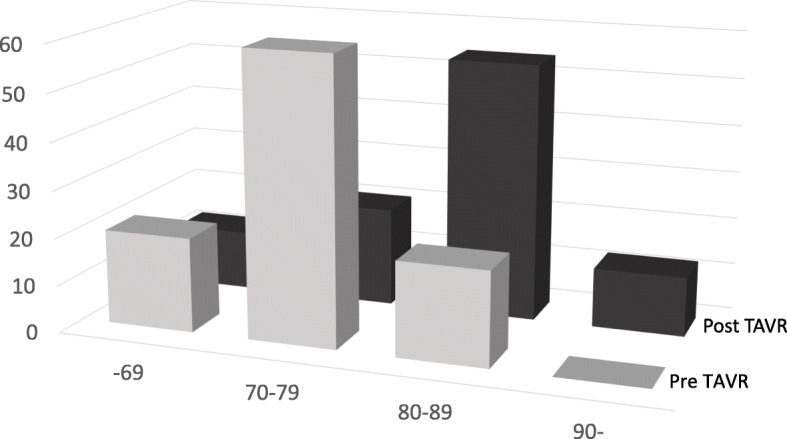
Age distribution

Age distribution is shown in Fig. 2. In the pre-TAVR group, 20% of patients were in their 80s and no patient was aged over 90 years. However, in the post-TAVR group, 54% of patients were in their 80s and 4.6% were aged over 90 years.

**Fig. 3:**
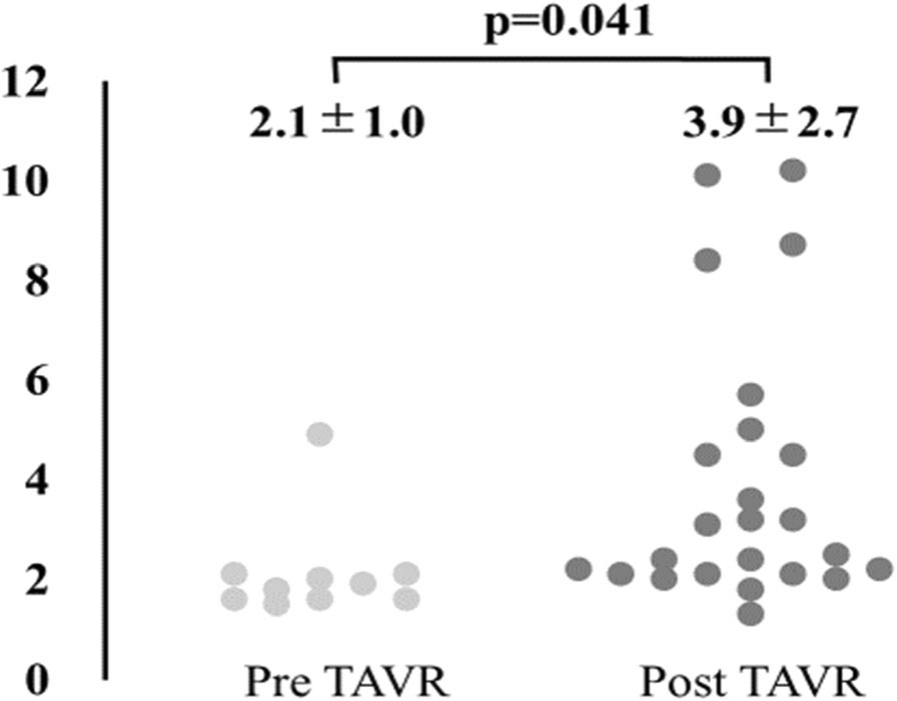
Japan score

Patient profile in the two groups is shown in Table 1. Patients in the pre-TAVR group were significantly older and had a smaller body surface area than those in the post-TAVR group. Japan score was significantly higher in the post-TAVR group than in the pre-TAVR group, and 17% of patients in the post TAVR group had a Japan score of greater than 8% (Fig. 3). Although there was no statistical significance in presence of cardiac symptoms, more patients were diagnosed as greater than New York Heart Association class II in the post-TAVR group. In the post-TAVR group, 45% of patients underwent TAVR.

**Table 1:**
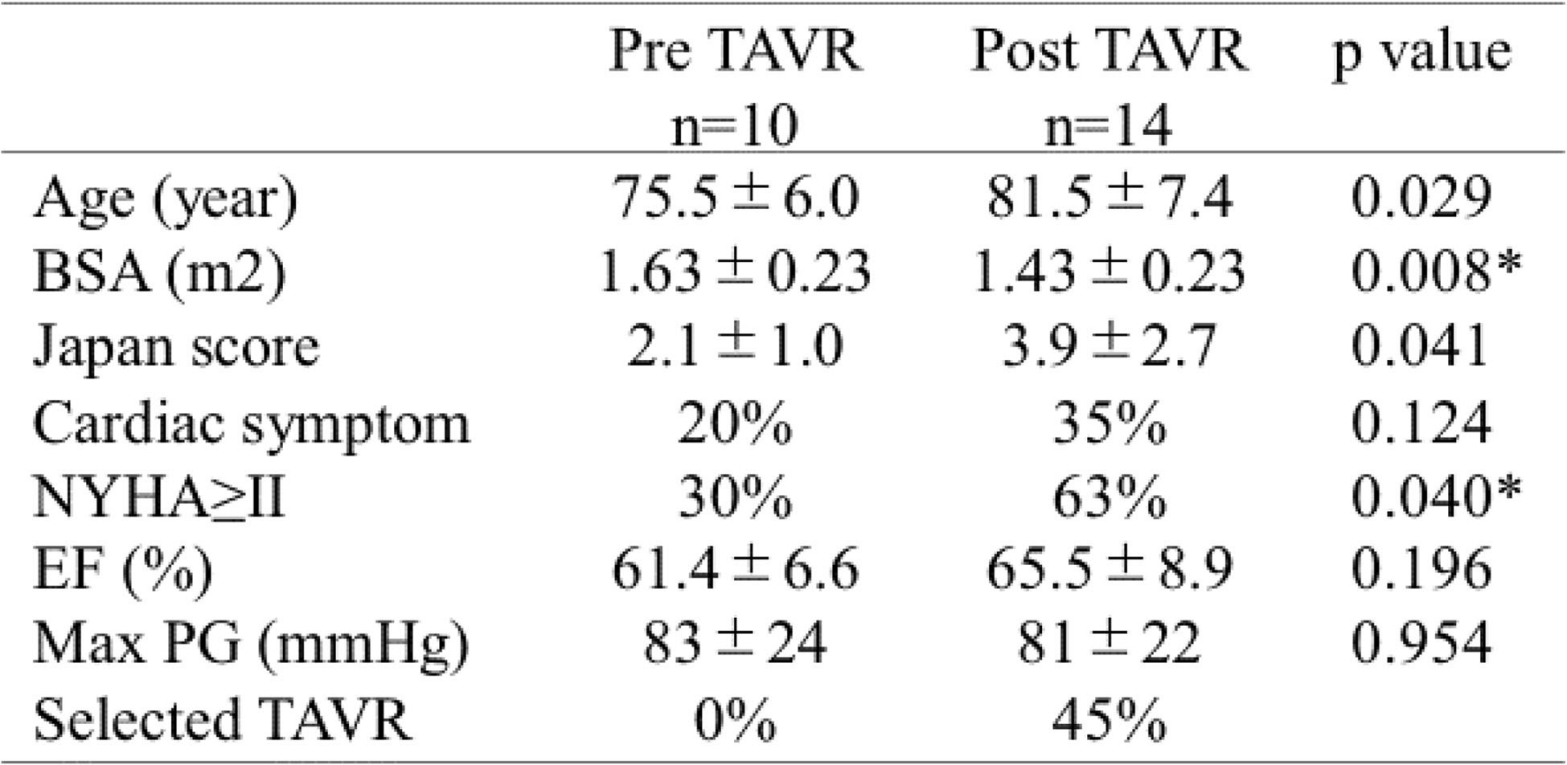
Patient’s profile in the pre-TAVR and post-TAVR groups

**Fig. 4:**
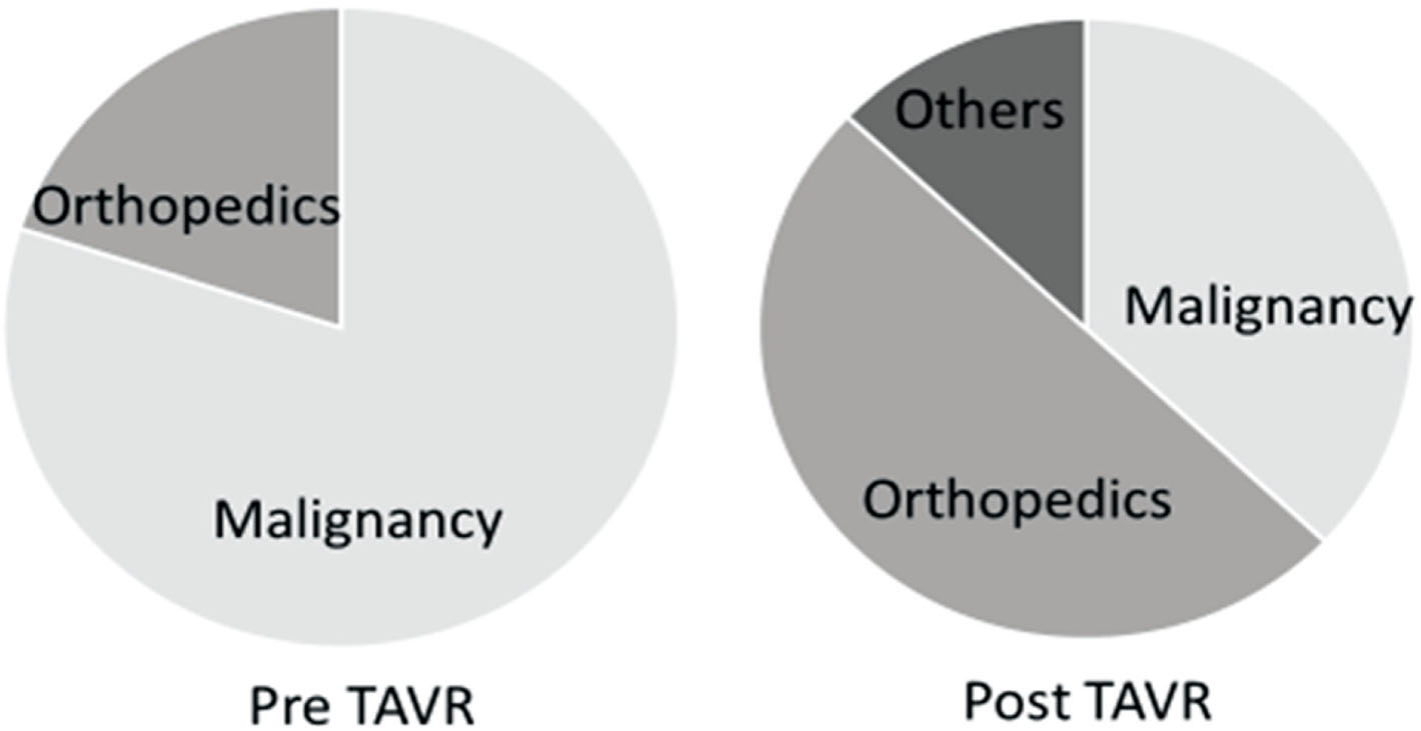
Non-cardiac disease

Diagnoses for non-cardiac surgery were malignancy in 8/10 (80%) and orthopedic conditions in 2/10 (20%) in the pre-TAVR group, versus malignancy in 9/24 (38%), orthopedic conditions in 12/24 (50%) and others 3/24 (13%; acute cholelithiasis, rectal prolapse, and strangulation of the sigmoid colon) in the post-TAVR group (Fig. 4).

### Pre-AVR vs. post AVR vs. TAVR

Compared with the pre-AVR group, the TAVR group were older and had a smaller body surface area. No statistical difference was seen between the pre-AVR group and post-AVR group in age or body surface area (Table 2). However, both the post-AVR group and TAVR group had a higher Japan score than the pre-AVR group (*p* < 0.001 in both comparisons).

**Table 2:**
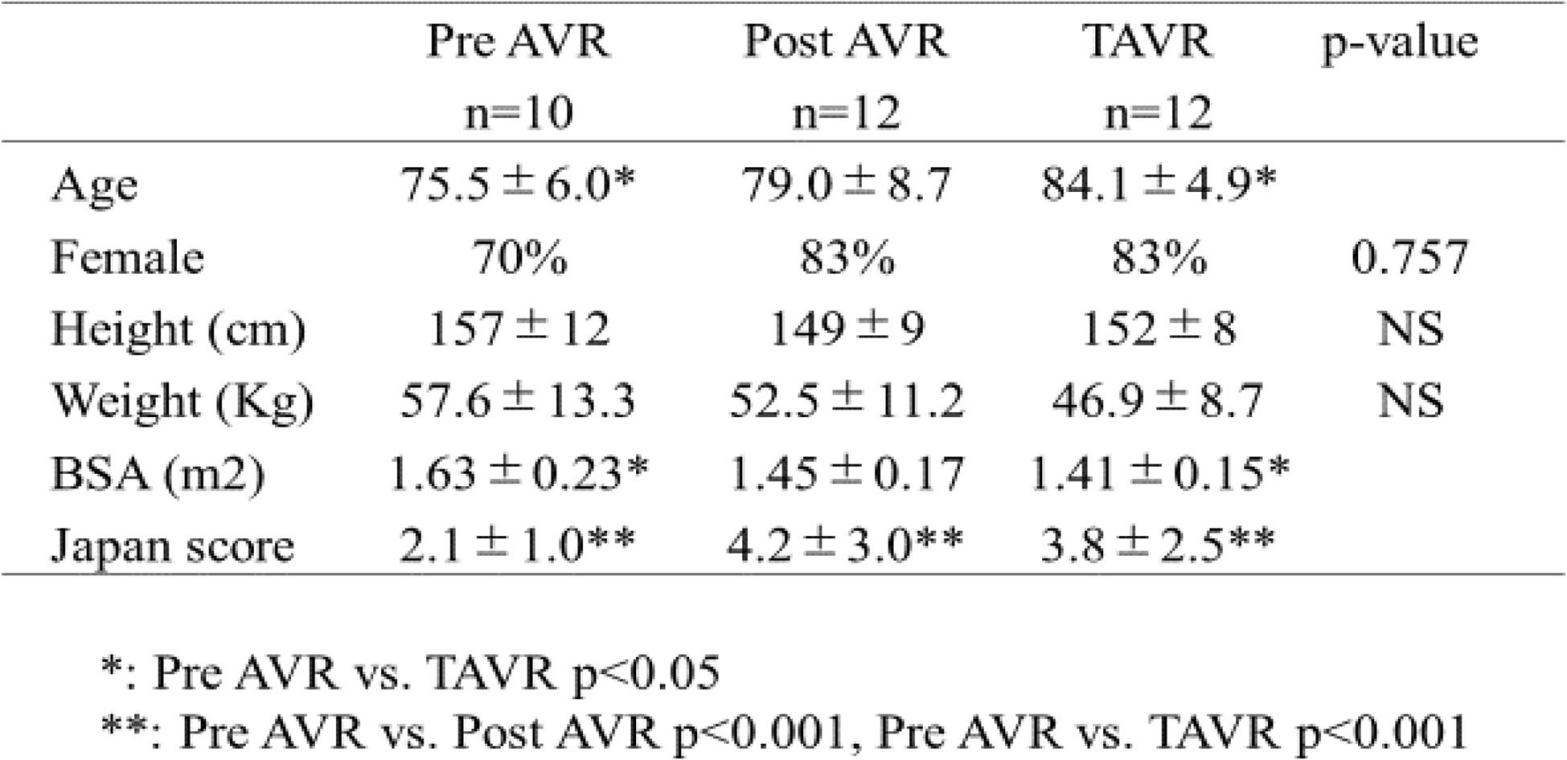
Patient profile in the pre-AVR, post-AVR and TAVR groups

**Fig. 5:**
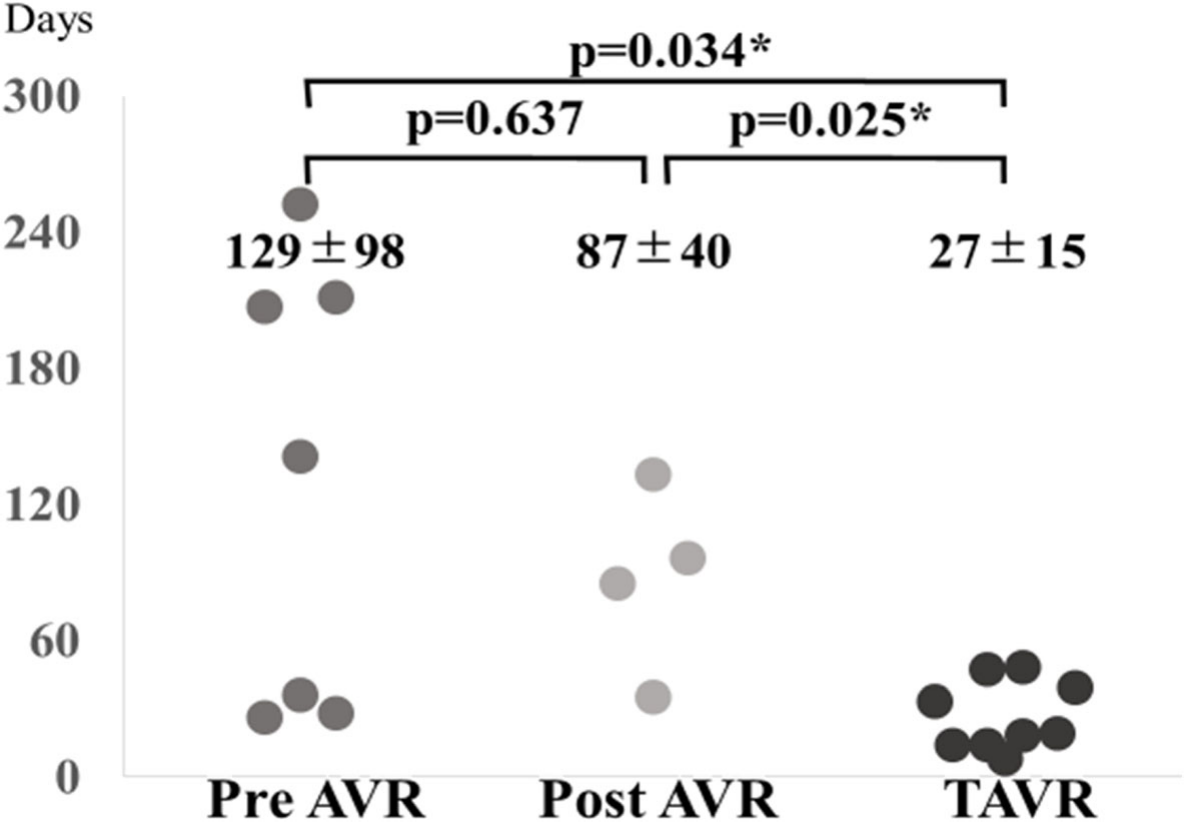
Interval between two operations

Completion rate of the two operations in the three groups was 70% in pre-AVR, 33% in post-AVR, and 75% in TAVR groups. This difference in completion rate was statistically significant between the TAVR and post-AVR groups (*p* = 0.010). Interval period between the two operations is shown in Fig. 5. The TAVR group had a shorter interval than both the pre-AVR and post-AVR groups. Also, all patients in the TAVR group underwent the second operation within 60 days after TAVR.

The reason for AVR selection in the post-AVR group was low risk for AVR in 7 patients, and anatomically unfit for TAVR in 5 patients, of whom 3 patients required a concomitant cardiac procedure (two-vessel coronary artery bypass in 1, and mitral valve replacement in 2), and 2 patients were on chronic hemodialysis (HD). Completion rate of non-cardiac surgery was 57% when AVR was selected due to low risk; however, no patient underwent non-cardiac surgery when AVR was selected due being in an unfit condition for TAVR (*p* = 0.081).

## Discussion

Patient age appeared to become older after the introduction of TAVR. Around 60% of the post-TAVR group were older than 80 years, and orthopedic disease became the most important non-cardiac condition after the introduction of TAVR. This phenomenon is understandable because both AS and orthopedic diseases, like degenerative arthritis disease, are related to aging. Before the TAVR era, many patients with severe AS who considered orthopedic operations might have abandoned the AS surgery without referring to cardiac surgeons.

We used the Japan score to evaluate operative risk. Although the Society of Thoracic Surgeons (STS) score in the United States and the European System for Cardiac Operative Risk Evaluation (EuroSCORE) II are useful for evaluating operative risks, their application to Japanese patients might be inappropriate. The Japan score was developed to establish an original Japanese risk predictive model [5]. Yamaoka et al compared the predictive value of operative mortality between EuroSCORE II, STS score and Japan score in patients undergoing AVR for AS [6]. Operative mortality was 3.4%, while the EuroSCORE II, STS score and Japan score were 3.1, 4.9 and 3.2%, respectively.

In our study, surgical risk of non-cardiac surgery with severe AS increased after the introduction of TAVR, as reflected by increased age and co-morbidities. There was an almost two-fold increase in Japan score after the introduction of TAVR, and 17% of patients a Japan score of more than 8%. As a result, there was a patient subgroup who should have undergone AVR due to their being unfit for TAVR, even though TAVR was preferable in the light of co-morbid conditions or frailty.

Three representative conditions render a patient unsuitable for TAVR. First, some patients are anatomically unsuited for TAVR. Examples include some bicuspid valves with a calcified raphe or an unfavorable calcified pattern, and patients with extreme aorta pathologies or diffuse peripheral arterial disease. Second, patients with significant mitral valve disease may develop heart failure during or after non-cardiac surgery if treated only by TAVR. Greater than moderate mitral regurgitation [7] was associated with an adverse long-term outcome after TAVR. Mitra Clip concomitant with TAVR [8] is an alternative option, but requires additional clinical studies to confirm its safety and appropriateness. Third, patients with HD are unsuitable for TAVR. Recent reports on TAVR in HD patients showed high early mortality [9, 10], and reimbursement for TAVR in HD patients has not been approved in Japan. However, TAVR may be considered if the durability and effectiveness of prosthetic valves in HD patients is demonstrated.

The role of TAVR in patients undergoing non-cardiac surgery is to reduce operative risk during and after non-cardiac surgery, and also to shorten the interval between the two operations. In the PARTNER 3 trial, patients who underwent TAVR had more rapid improvement in NYHA class and 6-min-walk than those who underwent AVR, which seems to be a valuable advantage in patients planning for non-cardiac surgery [11]. In patients with malignancy, a short interval between the two operations is particularly preferred due to disease progression. Further, TAVR may have some advantages in avoiding the spread and growth of cancer cells caused by cardiopulmonary bypass [12]. However, further studies of patient selection for TAVR are required, particularly for younger patients with malignancy.

## Conclusions

After introduction of transcatheter aortic valve replacement (TAVR), more senile and high-risk patients became candidates for two-staged operation, and orthopedic conditions became the most common non-cardiac disease. TAVR showed an advantage in shortening the interval between the two operations. The presence of patients whose condition makes them unfit for TAVR is a crucial problem, and future innovations in transcatheter valvular interventions and an expansion of indications are expected.

## Data Availability

Not applicable.

## Abbreviations

AS: Aortic stenosis
ACC: American College of Cardiology
AHA: American Heart Association
AVR: Aortic valve replacement
TAVR: Transcatheter aortic valve replacement
Pre-TAVR: Patients before introduction of transcatheter aortic valve replacement
Post-TAVR: Patients after introduction of transcatheter aortic valve replacement
Pre-AVR: Patients who underwent aortic valve replacement before the introduction of aortic valve replacement
Post-AVR: Patients who underwent aortic valve replacement after the introduction of aortic valve replacement
HD: Hemodialysis
EuroSCORE: European System for Cardiac Operative Risk Evaluation
STS: Society of Thoracic Surgeon
